# Benzo(a)pyrene parallel measurements in PM_1_ and PM_2.5_ in the coastal zone of the Gulf of Gdansk (Baltic Sea) in the heating and non-heating seasons

**DOI:** 10.1007/s11356-018-2089-9

**Published:** 2018-05-05

**Authors:** Anita Urszula Lewandowska, Marta Staniszewska, Agnieszka Witkowska, Magdalena Machuta, Lucyna Falkowska

**Affiliations:** Institute of Oceanography, University of Gdansk, Al. Marszałka J. Piłsudskiego 46, 81-378 Gdynia, Poland

**Keywords:** Benzo(a)pyrene, Parallel measurements, PM_1_, PM_2.5_, Heating and non-heating season, Baltic Sea coast

## Abstract

Parallel measurements of PM_1_ and PM_2.5_ aerosols were conducted in the urbanized coastal zone of the southern Baltic Sea. The main aim of the research was to assess and determine annual, seasonal (heating and non-heating), and daily concentration variability of benzo(a)pyrene in aerosols, these being the most dangerous constituents to human health. The average annual concentration of benzo(a)pyrene (B(a)P) was equal to 2.6 ng·m^−3^ in PM_1_ and 4.6 ng·m^−3^ in PM_2.5_, and both values were several times higher than the level of 1 ng·m^−3^ which was set out in the CAFE Directive. High mean daily concentrations of B(a)P persisted for 50 and 65% of the study period in PM1 and PM2.5, respectively. In order to determine the sources of B(a)P in both aerosol fractions, organic (OC) and elemental (EC) carbon concentrations were examined. The highest concentrations of all carbon species were reported during the heating season under local or regional land advection and at low air temperatures. The origin of pollutants was the same and was primarily related to the combustion of fossil fuels in the communal-utility sector. During the non-heating period, the role of transportation, both land and marine, increased and may have been significant in creating higher concentrations of carbon compounds in PM_1_ and PM_2.5_. Regardless of the size of the aerosol fractions, B(a)P loads introduced into the Baltic coastal zone were several times higher during the heating period compared to the non-heating season.

**Graphical abstract:**
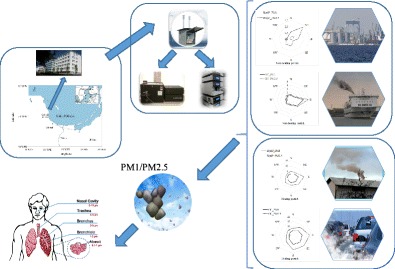
ᅟ

ᅟ

## Introduction

A considerable fraction (up to 50–70%) of atmospheric aerosol consists of carbonaceous material (Matta et al. 2003; Kim et al. 2012; Duhl et al. 2014). This can be emitted directly into the atmosphere by combustion of fossil fuels and biomass burning, as well as by non-combustion processes (e.g., biological particles, plant fragments, and soil-derived humic material). The direct emission of carbonaceous particles results in the production of primary aerosols, while secondary organic aerosols are formed from precursor gases by chemical reactions in the atmosphere (Satsangi et al. 2012). Particulate carbon is most often present in the form of (a) organic carbon (OC), a scattering material which contains carbon associated with hydrogen, and (b) elemental carbon (EC), a light-absorbing material with a graphite-type crystalline structure (Schauer et al. 2003; Sánchez de la Campa et al. 2009). While EC is generally of primary origin (traffic, industry, domestic heating, refuse burning, etc.), OC is a mixture of aliphatic and aromatic organic compounds including hydrocarbons, aldehydes, ketones, alcohols, and carboxylic acids, which may be derived from any of the aforementioned primary and secondary sources (Molnar et al. 1999; Lewandowska et al. 2010). In the past two decades, a special interest has developed among scientists in the occurrence of polycyclic aromatic hydrocarbons (PAHs) in aerosols. The most important of them is B(a)P, a product of incomplete combustion processes, due to the use of coal and wood for domestic heating purposes and transport-related emissions (petrol combustion, tarmac/asphalt, tire wear) (Ravindra et al. 2008; Schummer et al. 2014). Other sources of B(a)P, such as heavy industry, electric and coke plants, factories, combined heating and power plants, uncontrolled fires, and waste incineration, can also be of significance (Ravindra et al. 2008).

Carbonaceous particles can affect the radiation balance of Earth, either in a negative (cooling) or a positive (warming) sense, depending on its single scattering albedo (Harrison & Yin 2008; Khan et al. 2010). They are also very important in the context of local air quality and human health, especially in urban areas (Vanos et al. 2015; Witkowska et al. 2016a). The main area of scientific interest concerns polycyclic aromatic hydrocarbons since they are mutagens and carcinogens, and may also cause acute health effects (Lighty et al. 2000). Most information on the occurrence and toxicity of PAHs is related to benzo(a)pyrene (B(a)P), the best documented of these substances for causing lung tumors in animals upon inhalation (Thyssen et al. 1981; Wickramasinghe et al. 2012). B(a)P has been identified by the International Agency for Research on Cancer (IARC) as a Class 1 carcinogen to humans and as such it is regarded as the major indicator of air pollution by PAHs. Due to its high hydrophobicity, this compound is usually adsorbed onto particles (soot and dust) present in the atmosphere. Up to 95% of B(a)P may be associated with smaller particles (< 2.5 μm) and only a few percent with coarse aerosols (Zhou et al. 2005; Ji et al. 2007; Ravindra et al. 2008; Staniszewska et al. 2013). B(a)P present in small particles is more dangerous for human health as it can reach not only the pulmonary alveoli, but also the circulatory system and vital organs (Halek et al. 2006). Epidemiological studies confirmed relationships between the concentration of toxic substances in small respirable particles and the frequency of cancer cases (i.e., lung cancer) (Nielsen et al. 1996; Jung et al. 2010). B(a)P adsorbed on particles of diameters less than 1 μm is especially dangerous. In addition, PM_1_ aerosols can remain in the atmosphere for days or weeks and thus be subject to long-range transboundary transport in the air (WHO 2013). In the central and eastern part of Europe benzo(a)pyrene is a pollutant of increasing concern because its concentrations are often above the threshold set to protect human health (Garrido et al. 2014; Rybak and Olejniczak 2014). It is a ubiquitous and unavoidable contaminant, especially in the urban environment (Wickramasinghe et al. 2012). Nevertheless, parallel measurements of B(a)P concentration in PM_1_ and PM_2.5_ aerosols are rare. For this reason, they are the subject of our interests and the main research goal described in this publication. In Poland, the annual average concentration of benzo(a)pyrene in PM_10_ aerosols often exceeds the value of 1 ng·m^−3^ deemed acceptable for EU countries (Directive 2008/50/WE). Furthermore, the forecasts are not optimistic and do not provide an acceptable level of achievement in the coming years. The highest concentrations of B(a)P in Poland occur during the heating season due to the use of coal and wood for domestic heating purposes. Almost 90% of heat in Poland is generated from coal, and its consumption is equal to 77 million tons per year (https://www.worldenergy.org; https://euracoal.eu, 2018), making Poland the second largest coal consumer in the EU. Combustion of fossil fuel is therefore the fundamental source of B(a)P in air. Additionally in Poland, different kinds of garbage are burnt in residential heating systems together with coal and, due to the lack of any exhaust gas treatment installations (Ćwiklak and Pyta 2006), this can also constitute a significant source of air pollution. Higher concentrations of B(a)P in aerosols during the heating season were also noted in Gdynia. The mean concentration of B(a)P measured in total suspended matter (TSM) in the heating season in 2008 was equal to 2.18 ng·m^−3^ in comparison to the non-heating season, when the concentration was 50 times smaller (mean 0.05 ng m^−3^) (Staniszewska et al. 2013).

In consideration of the above, one of our main research goals was to assess not just the daily variability but also the seasonal (heating and non-heating) variability of benzo(a)pyrene concentrations in PM_1_ and PM_2.5_. Parallel annual measurements of PM_1_ and PM_2.5_ allowed us to determine in what synoptic conditions an increase in the concentration of B(a)P over the urbanized coastal zone of the southern Baltic Sea occurs. In order to identify the source areas of B(a)P, organic (OC) and elemental (EC) carbon concentrations were examined in both aerosol fractions. Year-round parallel measurements conducted in Gdynia showed how significant the problem is with regard to high concentrations of B(a)P in small aerosols and what percentage in PM_2.5_ constitutes toxic B(a)P adsorbed on PM_1_. This type of complex information on carbonaceous compounds measured simultaneously in PM1 and PM_2.5_ is not provided on a routine basis at the majority of monitoring sites, including the regional background air quality network of the UNECE/LRTAP/EMEP (European Monitoring and Evaluation Programme). For this reason the results obtained as part of our research may be useful in terms of setting daily concentration limit values for these small particles, which are known to be among the most dangerous to human health. For the moment, there are no such standards.

## Materials and methods

### Sampling

**Fig. 1:**
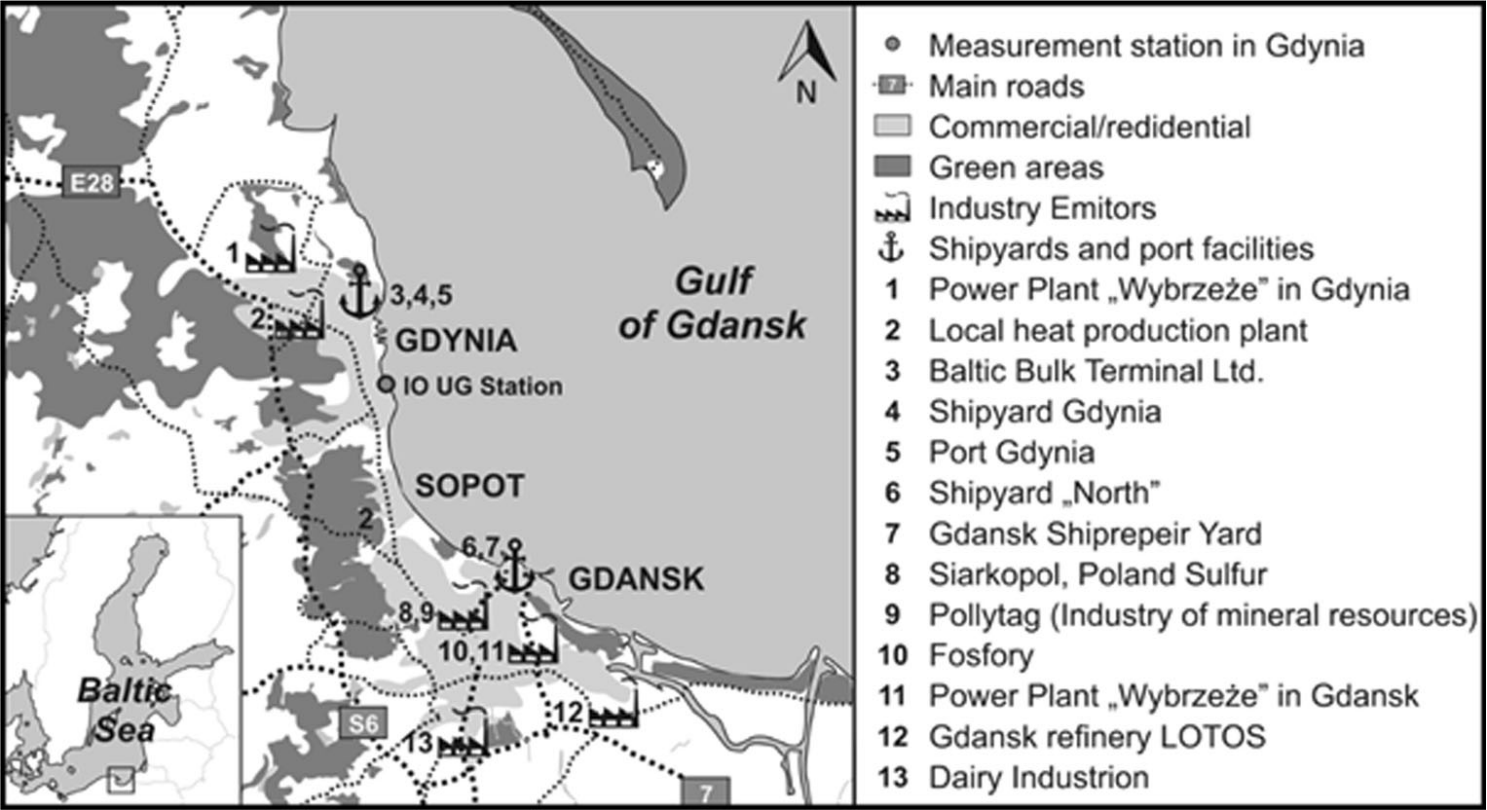
Location of the sampling station in Gdynia (IO UG) and surrounding emission sources

Samples of PM_1_ and PM_2.5_ aerosols were collected between 1 January and 31 December 2012 in Gdynia (*φ* = 54° 31′ N, *λ* = 18° 48′ E). The station is located on the roof of a six-story building belonging to the Oceanography Institute of Gdansk University (20 m above ground level). It is situated in the city center at a distance of about 800 m from the Gulf of Gdansk (Fig. 1). Gdynia is a large city with more than 250,000 inhabitants (http://stat.gov.pl/en/2012). The city is both commercial and residential, and there is urban and industrial development in all directions from the station. The site is located 300 m away from the largest commercial street in Gdynia, which is characterized by intense traffic (between 37,000 and 45,000 per day, www.bdl.stat.gov.pl) and high buildings on either side, creating a canyon-like arrangement. Two kilometers southwest of the station, there is a ring road, which serves as a thoroughfare for both personal and heavy-goods vehicles (Lewandowska and Falkowska 2013). In Gdynia, there is a harbor, a ship repair yard, and numerous industries including food, electronic, and paint production facilities, as well as construction (www.infoeko.pomorskie.pl). There are two other large cities in close proximity to Gdynia—Gdansk and Sopot. Altogether, the population of the “Tri-city” agglomeration amounts to nearly 1 million (http://stat.gov.pl/en/2012). Alongside urbanized areas, there are many forests. As much as 46% of this area is covered by woodland, most of which forms the Tri-city Scenic Park (www.infoeko.pomorskie.pl).

A low-volume (2.3 m^3^ h^−1^) HYDRA Dual Sampler (FAI Instruments) was used for parallel sampling of PM_1_ and PM_2.5_. Both aerosol fractions were collected in a 24-h cycle onto Pallflex Tissuqartz filters (47 mm diameter), which had been preheated at a temperature of 550 °C for a minimum of 6 h using a furnace, in order to eliminate volatile impurities and reduce blank values. After sampling, concentrations (in μg·m^−3^) of PM_1_ and PM_2.5_ were obtained by dividing the difference of filter weights (before and after sampling) by the volume of pre-filtered air. All procedures connected with weight measurements were performed with an accuracy of 10^−5^ g using the XA balance (RADWAG). Samples were stored in a desiccator at a temperature of 23 ± 2 °C and a relative humidity of 40 ± 5% (Lewandowska et al. 2010; Lewandowska and Falkowska 2013). The limit of quantification (LOQ) for PM_1_ and PM_2.5_ was 0.12 and 0.15 μg·m^−3^, respectively (for 20 replicates in every case). The uncertainty of the method was < 5.0% (at a certainty level of 99%).

### Chemical analysis

To determine the concentration of benzo(a)pyrene, a filter portion of 15 mm in diameter was used. The isolation of B(a)P from the collected samples was conducted by means of solvent extraction (3 cm^3^ acetonitrile: 1 cm^3^ dichloromethane 3:1 *v*/*v*) in an ultrasonic bath for 10 min at 21 °C (Periera et al. 2001; Halek et al. 2006; Staniszewska et al. 2013). B(a)P concentration was determined by means of liquid chromatography with a fluorescence detector (excitation, *λ* = 296 nm; emission, *λ* = 408 nm). The chromatographic separation was performed using a Nucleosil column (100-5 C18 PAH, 250 mm/4.6 μm) and mobile phase gradient (acetonitrile:water). The recovery of B(a)P in reference to the certified material (SRM-2585) amounted to 83%. The limit of quantification (LOQ) for the method was determined to be 0.01 ng·m^−3^ (Staniszewska et al. 2013).

For the purpose of organic and elemental carbon analysis in PM_1_ and PM_2.5_, a Sunset Laboratory Dual-Optical Carbonaceous Analyzer was used (EUSAAR 2 protocol). In order to perform the analysis, a section of filter measuring 1.5 cm^2^ was cut out, put in a quartz oven, and tested. Determination of the concentration of each carbon fraction was preceded by a series of analytical activities which, as with the analysis method itself, were conducted in accordance with the CEPA (2002) guidelines. The limit of quantification (LOQ) for measurement of OC was established at a level of 0.02 μg·m^−3^ while in the case of EC it was below LOQ and was therefore not taken into account in further calculations. The standard error of estimation was 6% for EC, and 10% for OC (with a certainty level of 99%). Blank samples were subtracted from the obtained results (Lewandowska et al. 2010; Lewandowska and Falkowska 2013). To calibrate the method, an external standard (analytically pure sugar solution) was used in addition to an internal standard. The analytical error of external calibration was on average 4.5%. Additionally, inter-laboratory comparison of the isotopic ^13^C method on Elemental Analyzer Instruments NC 2500 NC (with Université du Québec a Montréal) was performed. The agreement between the two methods was confirmed by a high Pearson correlation coefficient value for total carbon (*r* > 0.9) (Witkowska et al. 2016a).

### Characterization of meteorological conditions

Meteorological data (wind speed and direction, air temperature, and relative air humidity) was obtained using an automatic Milos 500 station by Vaisala. Data analysis indicates significant differences between the heating period (between January and March 2012 and between October and December 2012) and the non-heating period (between April and September 2012) (Table 1). The former was characterized by an inflow of cool and dry air masses, while during the latter the air masses were warmer and more humid. In both cases land advection of regional character prevailed. The proportion of marine air masses was higher in the heating period than in the non-heating period (38.0 and 17.0%, respectively). The direction of advection was determined for each measurement day, using the HYSPLIT NOAA model (Draxler and Rolph 2003; www.arl.noaa.gov/ready.html). A detailed description of the trajectories has been presented in previous papers (Lewandowska et al. 2010; Lewandowska and Falkowska 2013).

**Table 1:** Statistical characteristics of the meteorological conditions at the Gdynia coastal station during the measurement period (1 January–31 December 2012)

Period	Non-heating period^*e*^	Heating period^*f*^	Annual
$\overline{x}$T^*a*^±SD^*b*^ (min^*g*^- max^*h*^)	17.8 ± 3.7 (4.9–34.5)	3.7 ± 5.8 (−19.5–25.3)	7.7 ± 8.3 (−19.5–34.5)
$\overline{x}$Rh^*c*^±SD^*b*^ (min^*g*^- *max*^*h*^)	51.7 ± 10.4 (11.0–86.0)	60.6 ± 12.5 (11.0–90.0)	58.1 ± 2.6 (11.0–90.0)
$\overline{x}$*Vw*^*d*^±SD^*b*^ (min^*g*^- max^*h*^)	2.5 ± 0.7 (0.0–9.4)	3.2 ± 1.1 (0.0–27.1)	3.0 ± 1.1 (0.0–27.1)
Prevailing wind direction	17% sea 83% land	38% sea 62% land	28% sea 72% land

^a^average temperature [°C]

^b^Standard deviation

^c^average relative humidity [%]

^d^average wind speed [m s^−1^]

^e^Period between May and September 2012

^f^Period between January and April 2012 and between October and December 2012

^g^Minimal

^h^Maximal

### Statistical analysis

Statistical calculations of average, maximum, and minimum values as well as standard deviations were made using the STATISTICA® and EXCEL® programs. These were also used for the determination of linear regression coefficients, the standardization of data and the graphic presentation of results. In order to determine statistically significant differences in more than two sets of independent and nonparametric data, the Kruskal-Wallis test was used. For all dependencies presented in the publication, the level of significance (*p*) was established at less than 0.05.

## Results and discussion

### B(a)P concentrations in PM_1_ and PM_2.5_ aerosols in the atmosphere over Gdynia

The mean concentration of benzo(a)pyrene in PM_1_ was on a level of 2.6 ± 3.6 ng·m^−3^, and in PM_2.5_ it amounted to 4.6 ± 6.1 ng·m^−3^ (Table 2). However, the medians for B(a)P concentrations, amounting to 1.2 and 2.1 ng m^−3^ for PM_1_ and PM_2.5_ respectively, were twice as low as the mean. With the results proving nonparametric, the median seems to be more suitable to describe B(a)P concentrations as, among measurements of central tendency, it is the best representation of the middle value of the set of numbers (S-W: *p* = 0.00 for PM_1_ and PM_2.5_). Regardless of the form of data presentation, B(a)P concentration measured in Gdynia in 2012 was high and exceeded 1 ng m^−3^, both in PM_1_ and PM_2.5_ (Table 2). Benzo(a)pyrene is considered to be a representative of the group of polycyclic aromatic hydrocarbons. According to the WHO guidelines, its average annual concentration in PM_10_ should not exceed 0.12 ng m^−3^ (Garrido et al., 2014). In Directive 2008/50/EC issued by the European Parliament and according to the Council of 21 May 2008 on *Ambient Air Quality and Cleaner Air for Europe*, the permissible annual average concentration of B(a)P in PM_10_ aerosols is 1 ng m^−3^. For particles other than PM_10_, no limit values for B(a)P have been set to date. Hence, it was necessary for our results to be presented in relation to the value assigned to PM_10_. Measurements conducted in Gdynia in 2012 showed very high average annual concentrations of B(a)P, both in PM_1_ and PM_2.5_ (Table 2). The concentration of B(a)P was over 2.5 times higher in PM_1_ and more than 4.5 times higher in PM_2.5_ than the values allowed in PM_10_ by Directive 2008/50/EC (1 ng m^−3^). If WHO recommendations were taken into account (0.12 ng m^−3^), exceedances would have been up to 20 times higher for PM_1_ and 40 times higher for PM_2.5_. In 2012 in the atmosphere over Gdynia B(a)P in PM1 constituted on average 57% of B(a)P in PM_2.5_ aerosols mass. Previous measurements showed that PM_2.5_ represented up to 80% of PM_10_ mass (Witkowska et al. 2016b). Such results are alarming and highlight that special care must be taken for the effective protection of human health in the region of the southern Baltic Sea. In terms of the potential harmful influence of benzo(a)pyrene, its status as a carcinogenic compound means that it is more dangerous in PM_2.5_ and especially in PM_1_ aerosols, which penetrate into the lungs and bloodstream respectively (Kim et al. 2013; WHO 2013; Zwozdziak et al. 2016), than in PM_10_. Concentrations of B(a)P in particles smaller than 2.5 in diameter should therefore be as low as possible.

**Table 2:** Statistical characteristic of benzo(a)pyrene [ng·m^−3^], organic carbon [μg·m^−3^], and elemental carbon [μg·m^−3^] concentration in PM_1_ and PM_2.5_ at the Gdynia coastal station during the measurement period (1 January–31 December 2012)

Parameter		Estimator	Annual	Heating period^*a*^	Non-heating period^*b*^
PM_1_	PM_1_	$\overline{x}$ ^*c*^ ± SD	27.5 ± 12.4	31.5 ± 13.4	25.2 ± 9.7
		Med. ± (Q_1_–Q_3_)^*d*^	26.1 ± (19.4–33.9)	26.6 ± (20.3–35.1)	25.1 ± (18.4–32.0)
		Min–max	4.5–106.7	4.5–106.7	5.8–61.2
		*N*	296	196	100
	B(a)P	$\overline{x}$ ^*c*^ ± SD	2.6 ± 3.6	3.7 ± 3.9	0.2 ± 0.4
		Med. ± (Q_1_–Q_3_)^*d*^	1.2 ± (0.2–3.9)	2.3 ± (0.9–4.9)	0.1 ± (<0.1–0.2)
		Min–max	<LD^*e*^–22.1	<LD^*e*^–22.1	< LD^*e*^–1.9
		*N*	271	189	82
	OC	$\overline{x}$ ^c^ ± SD	5.8 ± 4.2	6.4 ± 4.8	4.5 ± 1.4
		Med. ± (Q_1_–Q_3_)^*d*^	4.7 ± (3.1–7.1)	5.3 ± (2.9–8.5)	4.3 ± (3.4–5.2)
		Min–max	0.5–33.3	0.5–33.3	1.2–7.8
		*N*	259	181	78
	EC	$\overline{x}$ ^*c*^ ± SD	1.9 ± 1.1	2.1 ± 1.2	1.5 ± 0.6
		Med. ± (Q_1_–Q_3_)^*d*^	1.6 ± (1.1–2.4)	1.8 ± (1.1–2.7)	1.4 ± (1.1–1.7)
		Min–max	0.4–6.1	0.4–6.1	0.4–3.7
		*N*	257	180	77
PM_2.5_	PM_2.5_	$\overline{x}$ ^*c*^ ± SD	32.8 ± 13.0	37.2 ± 14.0	29.8 ± 10.3
		Med. ± (Q_1_–Q_3_)^*d*^	30.9 ± (24.6–39.7)	31.8 ± (25.4–42.0)	28.8 ± (22.8–37.6)
		Min–max	7.6–109.4	8.5–109.4	7.6–68.1
		*N*	292	196	96
	B(a)P	$\overline{x}$^*c*^ ± SD	4.6 ± 6.1	6.3 ± 6.5	0.5 ± 1.0
		Med. ± (Q_1_- Q_3_) ^*d*^	2.1 ± (0.3–6.6)	4.4 ± (1.7–8.2)	0.1 ± (0.1–0.2)
		Min–max	<LD^*e*^–39.7	<LD^*e*^–39.7	<LD^*e*^–5.8
		*N*	258	183	75
	OC	$\overline{x}$ ^*c*^ ± SD	9.0 ± 6.6	10.2 ± 7.5	6.3 ± 2.1
		Med. ± (Q_1_–Q_3_)^*d*^	7.1 ± (4.7–11.0)	8.3 ± (4.7–13.6)	5.9 ± (4.8–7.7)
		Min–max	0.9–47.7	0.9–47.7	2.6–12.6
		*N*	243	169	74
	EC	$\overline{x}$ ^*c*^ ± SD	2.3 ± 1.3	2.5 ± 1.4	1.7 ± 0.8
		Med. ± (Q_1_–Q_3_)^*d*^	1.9 ± (1.3–2.9)	2.2 ± (1.4–3.4)	1.6 ± (1.3–2.2)
		Min–max	0.5–7.2	0.7–7.2	0.5–4.9
		*N*	241	167	74

^a^Period between May and September 2012

^b^Period between January and April 2012 and between October and December 2012

^c^Average value

^d^Median and Lower quartile-Upper quartile

^e^Limit of detection equal to 0.01 ng m^−3^

In 2012, the high concentrations of B(a)P in PM_10_ were characteristic for the whole of Poland, with the average value determined at 5.5 ng m^−3^ (GIOŚ 2013). According to the European Environment Agency (EEA), the average B(a)P concentration in Europe in 2012 ranged from 0.12 to 1.5 ng m^−3^ (http://www.eea.europa.eu). The EEA also estimated that in the past few decades there has been a reduction of around 47% in PAH emissions in Western Europe. In Poland, there is a general problem with the number of exceedances of the B(a)P target level (1 ng m^−3^). In 2012, it occurred at 107 out of 120 research stations (89%). The mean annual concentration of B(a)P at some sites exceeded the target by more than 1000% with the greatest values as high as 15 ng m^−3^ (GIOŚ 2013). In the following years, the situation even deteriorated. For example, in 2015 exceedances of the target level of B(a)P in PM_10_ occurred at 129 out of 136 stations (95%), with the annual mean concentration at some sites exceeding the target level by as much as 1600% (GIOŚ 2016). Our measurements conducted in Gdynia in 2012 confirmed the tendency towards deteriorating air quality in the region of the southern Baltic Sea. Concentrations of B(a)P in PM_1_ were higher than 1.0 ng m^−3^ for over 50% of the measurement time (142 times) and in PM_2.5_ for over 65% of the time (171 times). Previous results obtained for TSP in 2008 by Staniszewska and her co-authors (2013) indicated an annual average concentration of B(a)P which was a few times lower at a level of 1.3 ng m^−3^ (median 0.3 ng m^−3^) and exceedances of the target level were noted for just 7% of the measurement time.

In the atmosphere of urbanized cities like Gdynia, B(a)P can be easily adsorbed on particles smaller than 2.5 μm in diameter enriched with organic and elemental carbon, for which the origin is the same as B(a)P (Gupta and Kumar 2006). High concentrations of OC and EC in PM_1_ and PM_2.5_ could therefore have been expected in the atmosphere of Gdynia in 2012. However, their concentrations were typical and comparable to those noted at other urbanized stations located in Europe and also to those obtained in our previous studies (between 5 and 10 μg·m^−3^ for OC and between 1 and 2 μg·m^−3^ for EC) (Lonati et al. 2007; Lewandowska et al. 2010; Godec et al. 2012). The annual concentration of OC was 5.8 ± 4.2 μg·m^−3^ in PM_1_ and 9.0 ± 6.6 μg·m^−3^ in PM_2.5_. Mean concentration of EC was equal to 1.9 ± 1.1 μg·m^−3^ in PM_1_ and 2.3 ± 1.3 μg·m^−3^ in PM_2.5_ (Table 2). It was noticed that B(a)P and organic carbon had a common source of origin during the heating period (when *r* = 0.68 and 0.69 for PM_1_ and PM_2.5_, respectively; Spearman *p* < 0.05). This same tendency was noted for B(a)P and elemental carbon (*r* = 0.63 and 0.68 for PM_1_ and PM_2.5_, respectively; Spearman *p* < 0.05). In the non-heating period, when B(a)P concentrations were very low, such correlations were not obtained. In turn, the correlation between the concentrations of organic and elemental carbon in aerosols of both fractions was statistically significant both in the heating season (*r* = 0.82 and 0.79, for PM_1_ and PM_2.5_, respectively, Spearman *p* < 0.05) and the non-heating season (*r* = 0.64 and 0.54, for PM_1_ and PM_2.5_, respectively; Spearman *p* < 0.05), thus demonstrating their common source of origin regardless of the season. Detailed comparative characterization of OC and EC concentrations in aerosols collected in Gdynia in 2012 with those from other regions of the world is to be found in previous publications (Lewandowska et al. 2010; Witkowska et al. 2016a). The seasonal relationships between concentrations of B(a)P and organic and elemental carbon mentioned above prompted us to divide the data into two sets: heating and non-heating period.

### Factors determining B(a)P concentration in PM_1_ and PM_2.5_ aerosols over Gdynia in the heating and non-heating seasons

**Fig. 2:**
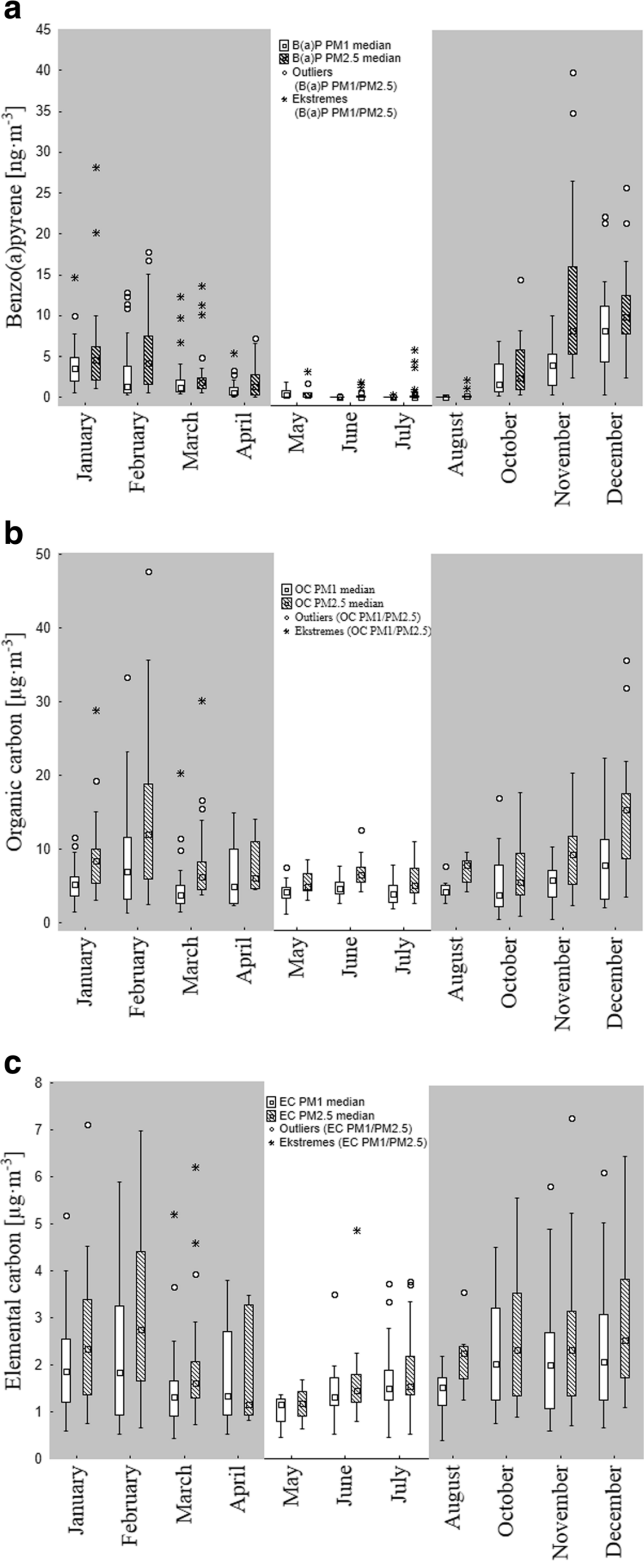
Monthly variability of **a** benzo(a)pyrene [ng m^−3^], **b** organic carbon [μg m^−3^], and **c** elemental carbon [μg m^−3^] concentration in PM_1_ and PM_2.5_ aerosols collected in 2012 in Gdynia. The gray sections of the graph represent heating months

In 2012, the highest concentrations of B(a)P in aerosols in Gdynia were noted during the heating period, which was considered to run from 1 October to 30 April. The non-heating season was between 1 May and 31 September. The median of B(a)P concentration was on average one order of magnitude higher during the heating period (2.3 ng·m^−3^ in PM_1_ and 4.4 ng·m^−3^ in PM_2.5_) than in the non-heating period (0.1 ng·m^−3^ in PM_1_ and PM_2.5_) (Table 2; Fig. 2a). The median of organic carbon concentration was also found to be higher in months with increased combustion for heating purposes (5.3 and 8.3 μg·m^−3^ in PM_1_ and PM_2.5_, respectively) than in non-heating months (4.3 and 5.9 μg·m^−3^, in PM_1_ and PM_2.5_ respectively) (Fig. 2b). A less clearly marked, although similar, dependence was found for elemental carbon. The median of its concentration was higher in the heating period (1.8 and 2.2 μg·m^−3^ in PM_1_ and PM_2.5_ respectively) than in the non-heating period (1.4 and 1.6 μg·m^−3^ in PM_1_ and PM_2.5_, respectively) (Fig. 2c). The non-parametric Kruskal-Wallis test for more than two groups of independent variables confirmed a statistically significant difference in the concentrations of B(a)P, OC, and EC in PM_1_ and PM_2.5_ between the heating and non-heating seasons of 2012 (*p* < 0.05).

**Fig. 3:**
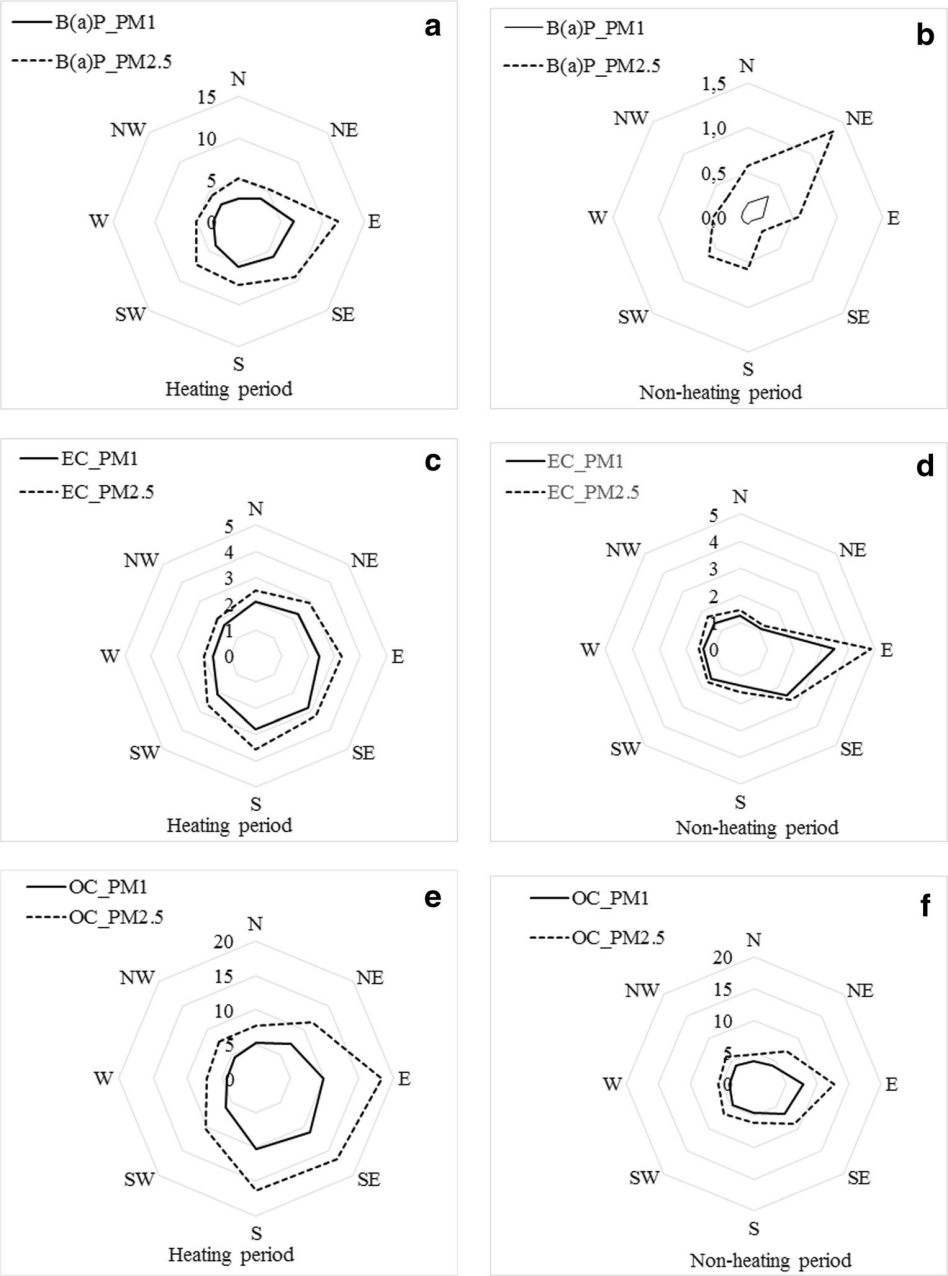
Variability of concentration of OC, EC [μg·m^−3^] and B(a)P [ng·m^−3^] in PM_1_ and PM_2.5_ aerosols collected in the heating and non-heating seasons of 2012 in Gdynia plotted according to air mass advection

The highest concentrations of all analyzed carbon compounds in the aerosols collected during the heating period in 2012 were as a consequence of low temperatures in the Tri-city region in the first and the last 2 months of 2012 and of low solar radiation (Table 2; Table 3). In January, the temperature fell to − 11 °C; in February, it was even as low as − 20 °C and in December − 12 °C. This led to an increase in carbon emissions as a result of increased household heating. In November, when the temperature was not as low as in the other months of the heating period (4.3 °C on average), the factor that determined high concentrations of carbon in aerosols could have been the use of individual home furnaces. In addition, at temperatures as moderate as those in November, the rate of PAHs photo-decomposition could have decreased with increased PAHs particle loading (Kamens et al. 1988). The increase of all carbon compound concentrations during the heating period also resulted from a low boundary layer during winter and weak dispersion of pollutants with low wind speed, on average equal to 3.2 m s^−1^ (Fig. 3; Fig. 1). Pollutants were transported with air masses drifting from over nearby habitations, moving from south to east, where many individual home furnaces are located (Fig. 1). At that time, the B(a)P/OC ratio was at its highest (7 × 10^−4^). In general during the heating period, the B(a)P/OC ratio was higher than in the non-heating period both in PM_1_ (6.0 × 10^−4^ and 0.2 × 10^−4^, respectively) and PM_2.5_ (6.0 × 10^−4^ and 0.9 × 10^−4^, respectively). The rate of emissions in domestic heating is greatly influenced by the nature of fuel (wood type, presence of foliage) and combustion conditions such as temperature, moisture, and availability of oxygen (Ravindra et al. 2008). Schauer and Cass (2000) attributed higher PAHs/OC ratios in PM_2.5_ aerosols during winter period measurements conducted in Los Angeles to the combustion of wood for domestic heating. Breivik et al. (2006) also suggested the residential sector as being a major source of PAHs emissions in recent decades. In the study area there are many flats and houses heated with hard coal, lignite or coke, often of dubious quality. A major additional problem is the combustion of waste in domestic furnaces (http://stat.gov.pl/en/2013). The combustion of fossil fuels in the communal-utility sector was the main source of carbon during the 2012 heating season, as confirmed by OC/EC ratios which ranged from 2.6 to 6.0 for PM_2.5_ (Pérez et al. 2008; Shen et al. 2010). In PM_1_ it was between 3.2 and 4.2.

**Table 3:** Monthly, annual, and seasonal (heating and non-heating) fluxes of benzo(a)pyrene introduced with PM_1_ and PM_2.5_ into the coastal zone during the measurement period (1 January–31 December 2012)

Period	B(a)P [μg·m^−2^·month^−1^]	
	PM_1_	PM_2.5_
January	45.7	65.7
February	27.4	45.7
March	25.5	31.5
April	12.7	11.8
May	1.8	12.0
June	0.4	2.6
July	0.3	4.6
August	0.6	0.7
October	9.0	17.4
November	19.0	35.6
December	36.7	83.1
Annual [μg m^−2^ year^−1^]	179.2	310.7
Heating period [μg m^−2^ season^−1^]	176.1	290.8
Non-heating period [μg m^−2^ season^−1^]	3.1	19.9

In the non-heating period of 2012, as much as 38% of B(a)P concentrations were below the LOQ (< 0.01 ng·m^−3^). Adsorption on highly porous, carbonaceous particles like soot or fly can provide some protection from B(a)P photo-oxidation in the warmer months of the year (Ravindra et al. 2008). However, during the non-heating period, the situation was slightly more complex than during the heating season. First and foremost, this was a time when the role of emissions originating from the combustion of fossil fuel for heating purposes was limited and the role of transportation increased, this period being one of standard enhanced harbor activity and heightened tourism in general. The emission of carbon species, including PAHs, from mobile sources is a function of engine type, load and age, fuel type and quality (e.g., aromaticity), PAHs accumulation in lubricant oil, lubricant oil combustion, and driving mode, including cold starting and emission control (Ravindra et al. 2008). Most studies show that the emissions from vehicle exhaust (diesel, leaded, and unleaded gasoline) are the largest contributors of PAHs in urban areas (Marchand et al. 2004; Marr et al. 2006; Ravindra et al. 2006a,b). In the non-heating period, B(a)P concentrations in PM_1_ and PM_2.5_ aerosols over Gdynia reached their highest values at advection from NE to E, where the sea harbor is located along with a shipyard and an access channel for vessels and ferries (Fig. 3; Fig. 1). The concentration of B(a)P in PM_2.5_ was then more than 2.5 times higher than at other directions of air mass inflow (1.3 and 0.5 ng·m^−3^, respectively) (Fig. 3b). In PM_1_, the concentration of B(a)P at advection from the port was as much as three times higher (0.3 ng·m^−3^ and 0.1 ng·m^−3^, respectively). This same increase in concentration under north to east advection was noted for OC and EC (Fig. 3c–f). The origin of carbon from mobile sources is most often described in literature as an OC/EC value below 2.5 (Shen et al. 2010; Titta et al. 2013). Under advection from the harbor, OC/EC ratios were equal to 2.2 for PM_1_ and 2.4 for PM_2.5_ and a statistically significant relationship was noted between B(a)P and EC concentrations both in PM_1_ (*r* = 0.64, *p* < 0, 05) and in PM_2.5_ (*r* = 0.75, *p* < 0.05). Apart from the Baltic Sea shipping traffic, a significant influence on the increase in B(a)P and other carbon species concentrations at North-East advection may have been exerted by the daily activity in the seaports (e.g., transshipment and consequent traffic or the re-suspension of pollutants deposited in docks) (Alastuey et al. 2006). The Port of Gdynia is one of the harbors of vital importance to the national economy and is thriving. Between 2000 and 2012 the transshipment rate increased by nearly 40% (www.port.gdynia.pl). Another issue is the emission of volatile organic compounds that undergo conversion into carbonaceous aerosols when vessels enter or leave the harbor. According to the International Convention on Marine Pollution of 1972, the fuel used on ships should be of good quality and contain no substances that adversely affect air pollution. When entering/leaving ports, ships normally use cleaner, high-octane fuel, and low-quality fuel is used only in the open sea. Nevertheless, high-octane fuel tends to wash pollutants out of engines, which at the stage of leaving ports can have a negative effect on the quality of air and may account for higher concentrations of the analyzed compounds at advection from the port, from North-East. The results presented above may indicate the role of sea transport in shaping high concentrations of B(a)P in aerosols; however, they require further research and careful analysis.

### The deposition of B(a)P with PM_1_ and PM_2.5_ aerosols into the coastal zone of the southern Baltic

Once aerosols containing carbon are released into the atmosphere, they undergo atmospheric transportation, degradation, and deposition to surface environments. In the case of PAHs, dry deposition dominates as they are hydrophobic and may be easily bound to particles suspended in the air. As much as 70% of B(a)P in wet precipitation was found to be adsorbed on aerosol particles (Ravindra et al. 2008). The measurements performed at the coastal station in the Baltic Sea in 2012 made it possible to determine fluxes of B(a)P introduced into the coastal zone with PM_1_ and PM_2.5_ particles. All fluxes (Ia) were estimated using the formula given by Seinfeld and Pandis (2012) and deposition velocity was calculated according to the formula by Slinn and Slinn (1980). The method for calculating fluxes of B(a)P deposition was described in previous papers (Lewandowska et al. 2010; Witkowska et al., 2016a).

The annual loads of benzo(a)pyrene were equal to 179.2 and 310.7 μg·m^−2^·y^−1^ for PM_1_ and PM_2.5_, respectively. Regardless of the size of the aerosols, the loads introduced into the Baltic coastal zone were several times higher during the heating period than in the non-heating period. The maximum value for B(a)P in PM1 was noted on January 6, 2012 (6.1 μg·m^−2^·d^−1^), and in PM2.5 on December 14, 2012 (14.8 μg·m^−2^·d^−1^). The high values of B(a)P on January 6 coincided with the Feast of Epiphany, a public holiday characterized by intensified traffic. In December, there was a drop in air temperature down to − 10 °C and a decrease in wind speed to 2 m·s^−1^. The increase in B(a)P fluxes at that time was caused by intensified emission of pollutants from the communal-utility sector for heating purposes and poor subsequent dispersion of those pollutants. Higher fluxes during the cold months of the year could lead to elevated concentrations of organic substances in surface seawater, resulting in an increase in the thickness of the sea microlayer and a decrease in the air-sea gas exchange and velocity of CO_2_ transfer. In situ measurements, as well as laboratory experiments, confirmed this rule (De Grandpre et al. 1995; Falkowska 1999). Our previous research also demonstrated that atmospheric deposition seems to be an important factor in the carbon enrichment of surface seawater and could explain up to 18% of total carbon flux (OC plus EC) in the coastal zone of the Southern Baltic region (Witkowska et al. 2016a and b). Determining the load of benzo(a)pyrene introduced into seawater from the atmosphere appears to be an extremely important task. Although most combustion-derived (pyrogenic) PAHs are deposited close to their source, atmospheric transport can carry a significant amount of these compounds to remote locations and PAHs may therefore be found in lake sediments, deep sea sediments, and even arctic ice and snow (Ravindra et al. 2008). In the case of the Baltic, a significant amount of PAHs are introduced into its sediments via the River Vistula (Staniszewska et al. 2011). However, Lubecki and Kowalewska (2010) have indicated that atmospheric deposition may also be an important source of polycyclic aromatic hydrocarbons. This source is of particular importance for PAHs with more than four aromatic rings. Due to their hydrophobicity, heavier PAHs accumulated in sediments are not mobile and may pose a greater threat to living organisms (Abdel-Shafy and Mansour 2016).

## Conclusions

In 2012, measurements of PM_1_ and PM_2.5_ aerosols were performed in order to determine the daily, seasonal (heating and non-heating) and annual variability of benzo(a)pyrene concentrations. The average annual concentrations of B(a)P turned out to be alarming for the health of the inhabitants of Gdynia and its surroundings.

In the smallest PM_1_ aerosols, those which most easily penetrate into the lungs, the annual values of B(a)P concentrations were more than 2.5 times higher, and in PM_2.5_ more than 4.5 times higher than the average annual B(a)P limit of 1 ng m^−3^ (CAFE Directive 2008/50/EC). The medians of annual B(a)P concentrations were also above the normative value, although significantly lower. In PM_1_ the B(a)P median amounted to 1.2 ng m^−3^, and in PM_2.5_ it was at a level of 2.1 ng m^−3^. Given the non-parametric nature of the obtained data, the median gives a better representation of the middle value of the set of numbers.

On the basis of the conducted measurements, it was found that the mean daily concentrations of B(a)P were also very high, persisting above 1 ng m^−3^ for 50% and 65% of the study time, for PM_1_ and PM_2.5_ respectively. These were mainly recorded during the heating season. At that time, the temperature dropped to very low values (as low as − 20 °C), the dispersion of impurities was poor owing to weak ventilation, and air masses were inflowing from residential areas. Parallel measurements of B(a)P, OC and EC in PM_1_ and PM_2.5_ made it possible to determine that in Gdynia the dominant source of carbon during the heating season were combustion processes in the communal-utility sector due to the use of coal and wood for heating purposes.

In the non-heating period, when combustion processes were limited, the predominant source of B(a)P in PM_1_ and PM_2.5_ aerosols was transportation. B(a)P concentrations reached two to three times higher values under advection from the harbor area. During the measurement period marine transportation, daily activity in the Port of Gdynia and usage of the access channel to the Port of Gdynia by ships and ferries, may have been responsible for a periodic increase in B(a)P concentration in aerosols.

In 2012, regardless of the size of the aerosol fractions, B(a)P loads introduced into the Baltic coastal zone were one to two orders of magnitude higher during the heating period than in the non-heating season, this being the result of higher B(a)P concentrations from intensified emission of carbon used for heating purposes.
